# Supplementary material for: Cerebro-cerebellar networks facilitate learning through feedback decoupling
Source: Nat Commun. 2023 Jan 4;14:51. doi: 10.1038/s41467-022-35658-8 (PMC9813152; doi:10.1038/s41467-022-35658-8)
Supplement: Supplementary file 1 — Supplementary Information [file 41467_2022_35658_MOESM1_ESM.pdf]

Supplementary Information

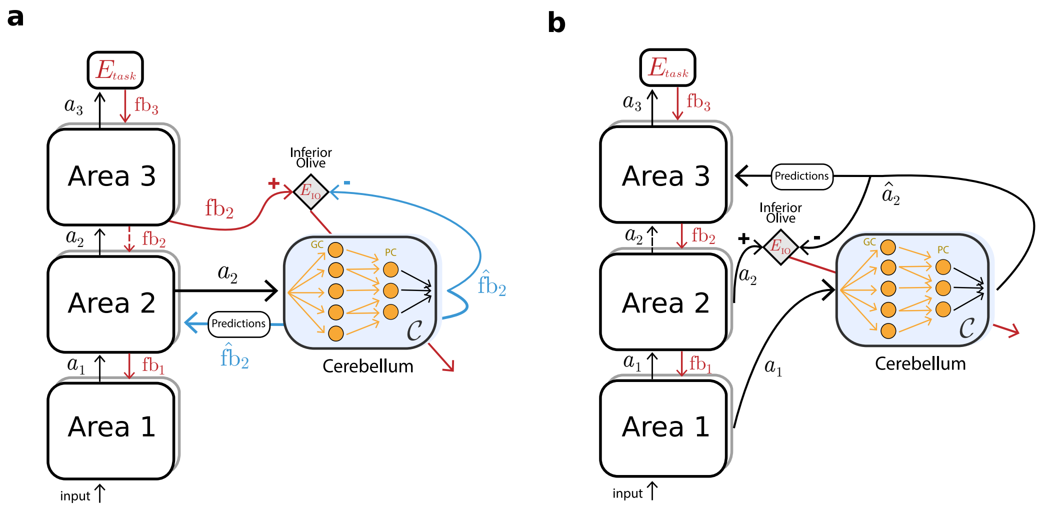

Figure S1. Cerebellum as decoupling machine in feedforward multi-area networks. (**a**) Illustration of decoupling feedback processing. The cerebellum makes predictions of the feedback expected by brain area 2, decoupling the main network from downstream brain areas (dashed red arrow). (**b**) Case of decoupling feedforward processing. The cerebellum predicts the forward activity expected by brain area 3, thereby approximating (and decoupling) the forward computations between brain area 1 and 3 (dashed black arrow). Note that the cerebellum could, in principle, approximate feedback and feedforward processing across many more brain areas (i.e. brain area 2 could be expanded in multiple brain areas).

Table S1. Relationship between the internal models of the cerebellum with decoupling machines[1]**.** The properties of the forward model of the cerebellum can be set against those of feedback decoupling (blue); similarly, the properties of the inverse model of the cerebellum can be set against those of forward decoupling (red). The internal models here focus on the classical motor control setting but can be extended to cognition, where for example a "mental model" replaces the "controlled object"[2]. Abbreviations: MM, main model; temp., temporal; spat. spatial.

	Forward Model	Feedback Decoupling	Inverse Model	Forward Decoupling
controller	cerebral (motor) cortex	main model (MM)	cerebellum	synthesiser
input	motor state/command	area state*	sensory/desired state	(temp.) area state*<br>(spat.) upstream state*
output prediction	future state	(temp.) future gradient<br>(spat.) downstream gradient	motor command	(temp.) future state<br>(spat.) downstream state
output destination	cerebral (motor) cortex	MM: same area	controlled object	(temp.) MM: same area<br>(spat.) MM: downstream area

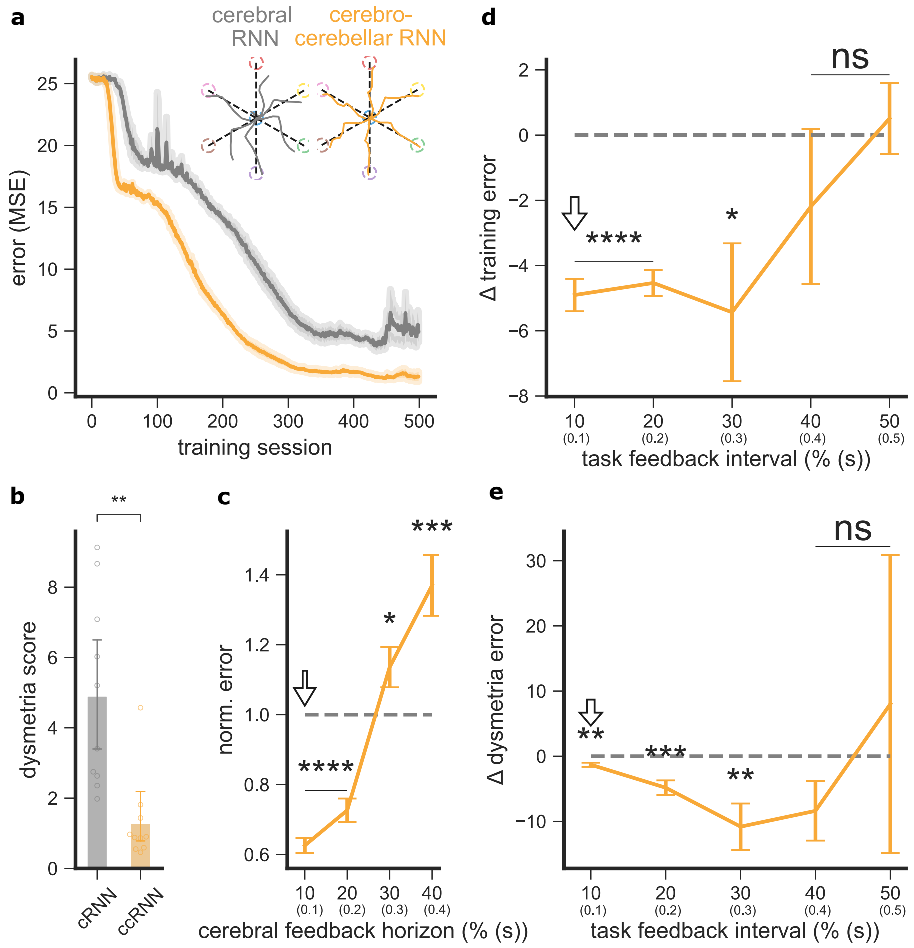

Figure S2. Cerebro-cerebellar model improves learning and output behaviour when using a point-mass model in the line drawing sensorimotor task. (**a**) Error between model output and desired target trajectories for cerebellar RNN (gray, cRNN) and cerebro-cerebellar RNN (orange, ccRNN) over learning. Insets: Model trajectory produced for all cues after learning. (**b**) Dysmetria score for cRNN and ccRNN. The dysmetria score quantifies how smooth the movement is after learning (Methods). (**c**) Normalized model mean squared error (MSE) after learning for different cerebral feedback horizons. Feedback horizon is denoted as percentage of the total task sequence. Arrow indicates feedback horizon used by the cerebral network in the other panels. (**d**) Difference in training error between for varying degrees of task feedback interval (ns denotes not significant: 0.394 (40%) and 0.661 (50%)). (**e**) Difference in dysmetria score between ccRNN and cRNN for varying degrees of task feedback intervals (ns denotes not significant: p=0.098 (40%) and 0.744 (50%)). Task feedback interval given as a percentage of the total task time. **: p<0.01, ***: p<0.001, ****: p<0.0001 (two-sided paired t-test). Error bars represent mean $\pm$ SEM across 10 different initial conditions. Source data are provided as a Source Data file.

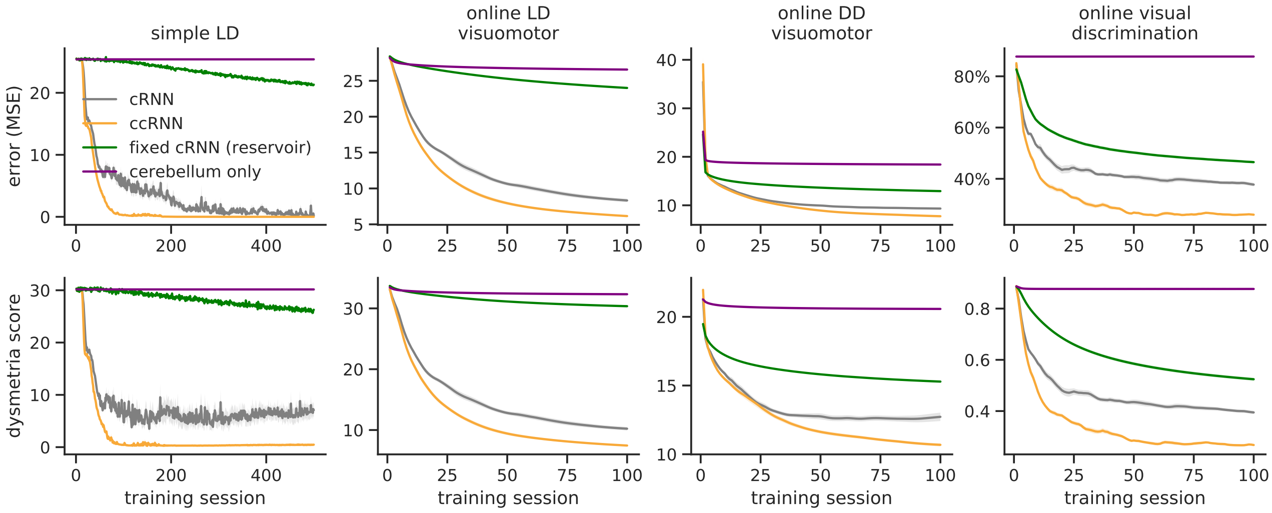

Figure S3. cRNN and ccRNN models compared to a fixed RNN with fixed weights (equivalent to a reservoir RNN) and a model with only the feedforward cerebellar network across tasks. For the lone cerebellar network there is no recurrency in the network at all and must directly translate the current external input to desired output; for the simple line drawing task which requires memory of the initial cue this removes the possibility of any learning at all (optimal case shown). Error bars represent mean ± SEM across 10 different initial conditions. Source data are provided as a Source Data file.

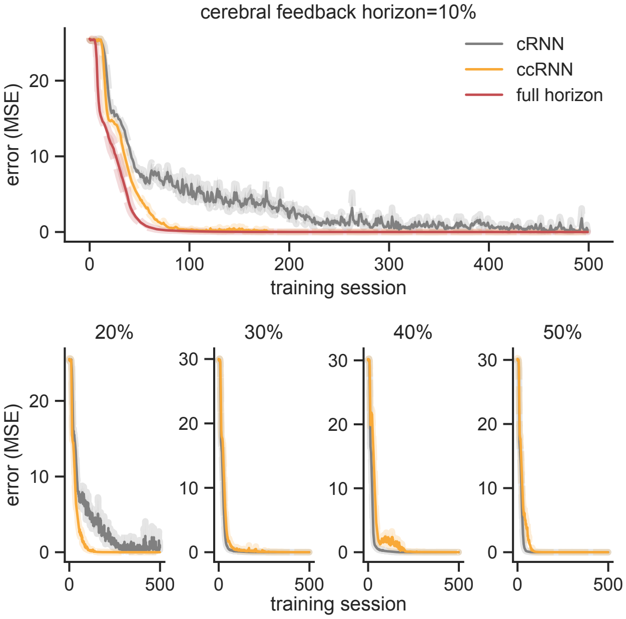

Figure S4. Learning for different cerebral feedback horizons for the line drawing task (cf. Fig. 2d). Feedback horizon is given as percentage of task duration (10 time steps). Results presented in main text (Fig. 2b) shown on top row along with RNN trained with full horizon (i.e. cerebral feedback horizon = 100%). Error bars represent mean ± SEM across 10 different initial conditions. Source data are provided as a Source Data file.

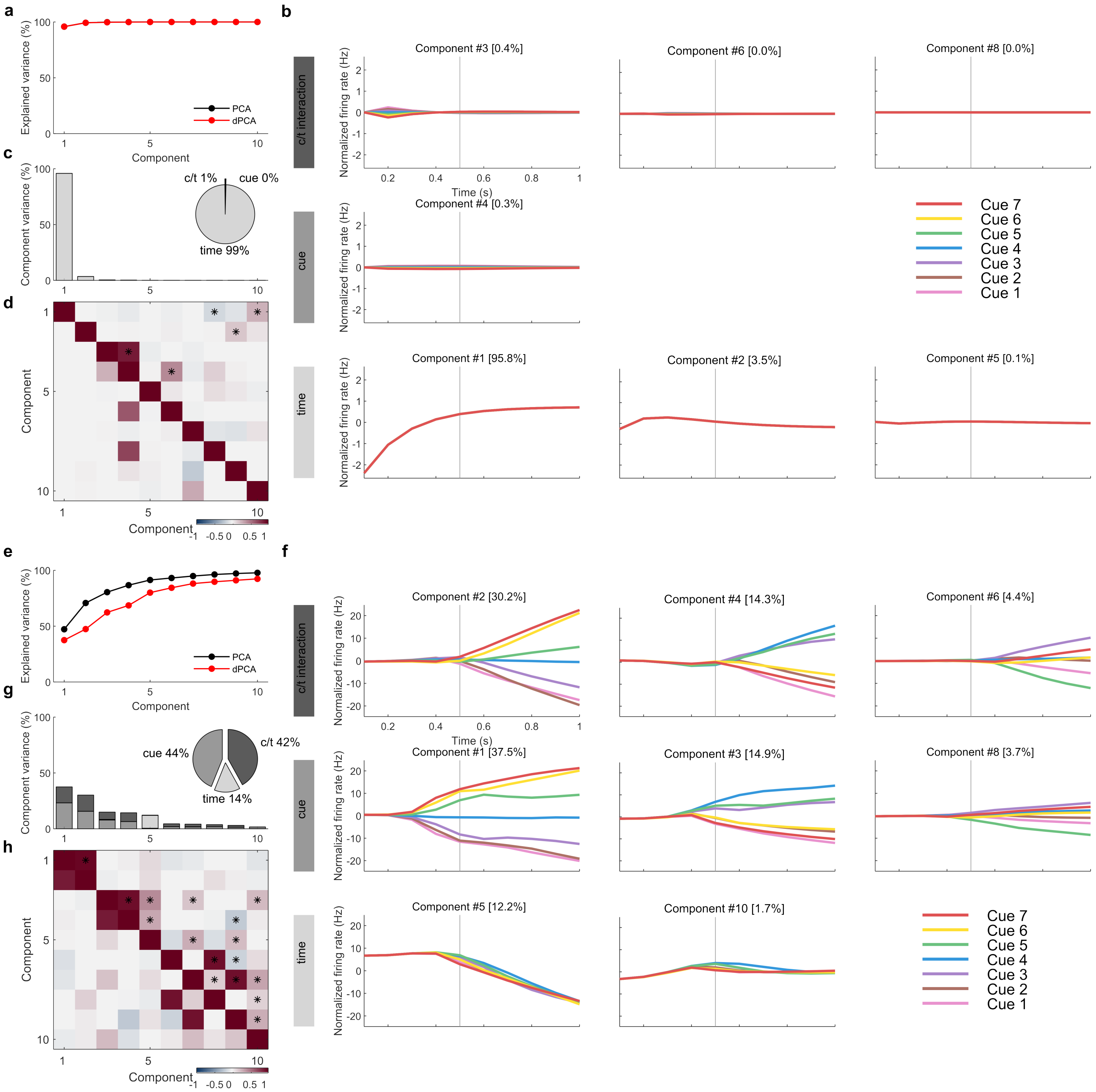

Figure S5. Demixed PCA of cRNN network at the beginning and end of learning (cf. Fig. 2e,f). Early and late learning corresponds to training session 1 (top a-d) and 200 (bottom e-h), respectively. (**a, e**) Cumulative variance explained by PCA (black) and dPCA (red) components. (**b, f**) Demixed principal components for cue, time and cue/time interaction task variables. In each subplot there are 7 lines corresponding to the 7 cues (cf. Fig. 2a). (**c, g**) Explained variance for individual demixed principal components. Pie chart shows how the total variance is split between different task variables. (**d, h**) Dot product between all pairs of the first 15 demixed principal components (upper-right triangle) and correlations between all pairs of the first 10 demixed principal components (bottom-left triangle). Stars denote statistical significance of the two PC axes being significantly non-orthogonal[3] (p<0.001, one-sided paired t-test for $f_1 \cdot f_2 > 3.3\sqrt{N}$, where f_1, f_2 are the respective axes and N the dimension of the the ccRNN cerebral network). Source data are provided as a Source Data file.

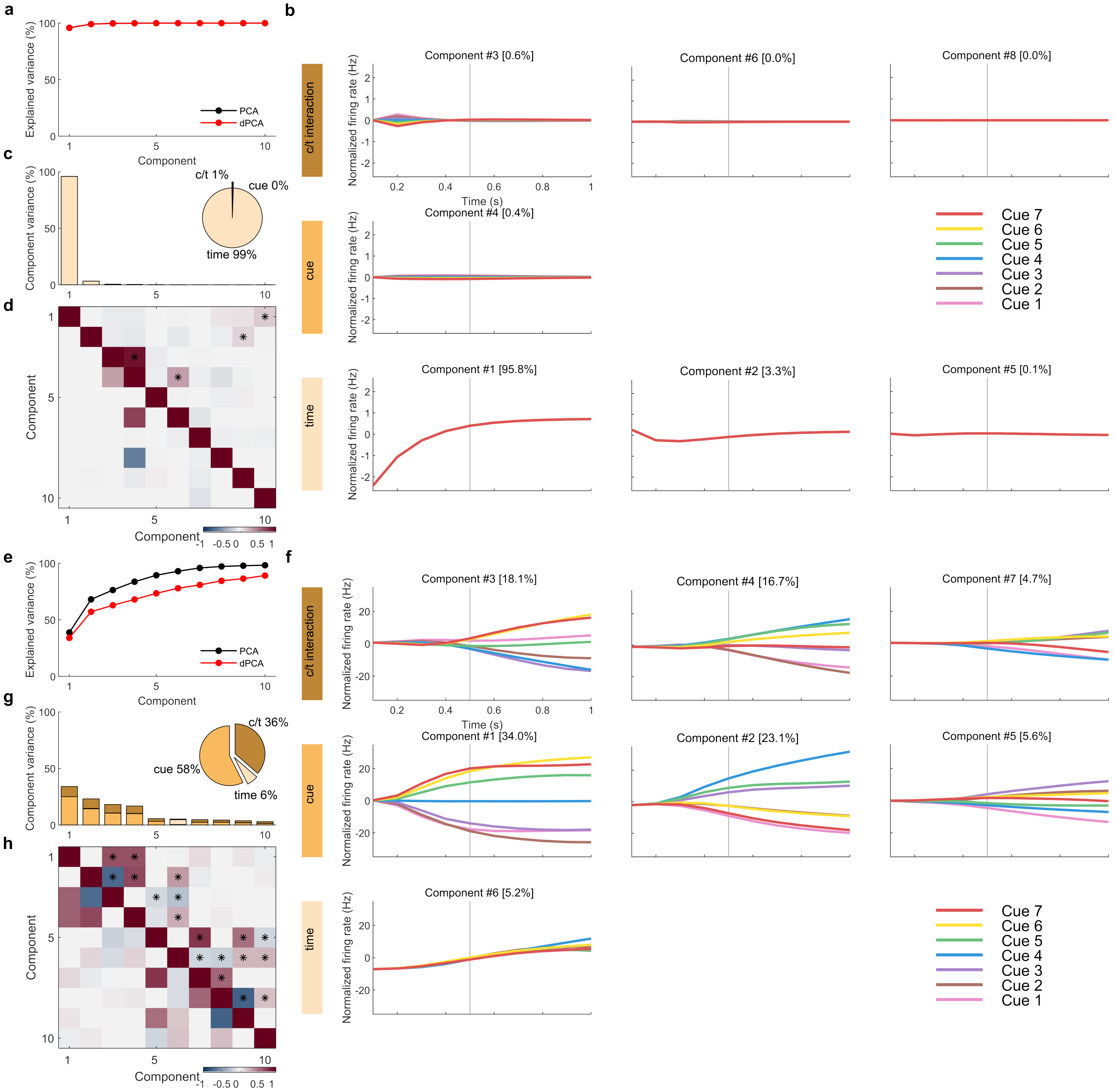

Figure S6. Demixed PCA of ccRNN cerebral network at the beginning and end of learning (cf. Fig. 2e,f). Early and late learning corresponds to training session 1 (top a-d) and 200 (bottom e-h), respectively. (**a, e**) Cumulative variance explained by PCA (black) and dPCA (red) components. (**b, f**) Demixed principal components for cue, time and cue/time interaction task variables. In each subplot there are 7 lines corresponding to the 7 cues (cf. Fig. 2a). (**c, g**) Explained variance for individual demixed principal components. Pie chart shows how the total variance is split between different task variables. (**d, h**) Dot product between all pairs of the first 15 demixed principal components (upper-right triangle) and correlations between all pairs of the first 10 demixed principal components (bottom-left triangle). Stars denote statistical significance of the two PC axes being significantly non-orthogonal [3] (p<0.001, one-sided paired t-test for $f_1 \cdot f_2 > 3.3\sqrt{N}$, where f_1, f_2 are the respective axes and N the dimension of the the ccRNN cerebral network). Source data are provided as a Source Data file.

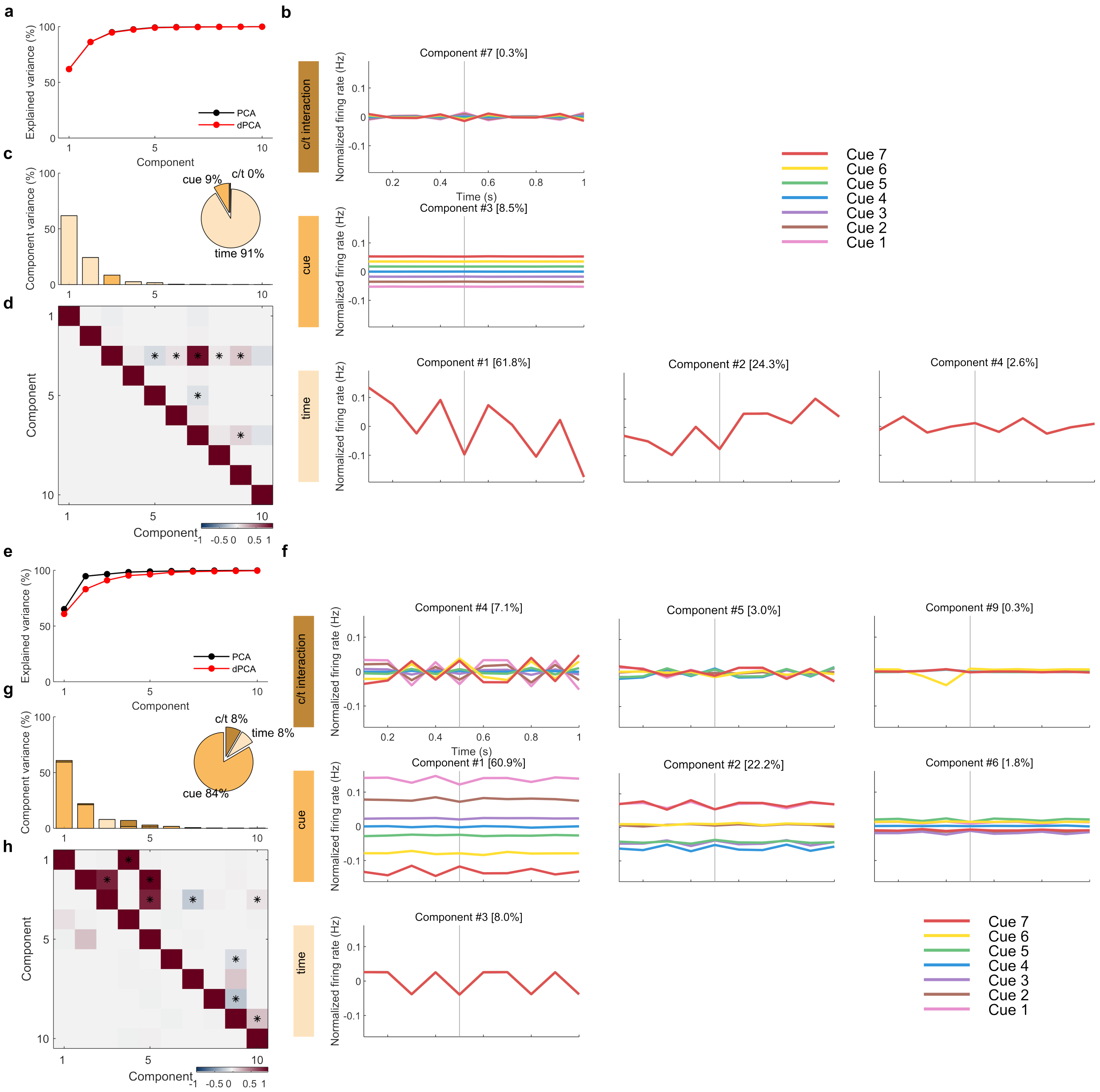

Figure S7. Demixed PCA of ccRNN cerebellar network at the beginning and end of learning (cf. Fig. 2g,h). Early and late learning corresponds to training session 1 (top a-d) and 200 (bottom e-h), respectively. (**a, e**) Cumulative variance explained by PCA (black) and dPCA (red) components. (**b, f**) Demixed principal components for cue, time and cue/time interaction task variables. In each subplot there are 7 lines corresponding to the 7 cues (cf. Fig. 2a). (**c, g**) Explained variance for individual demixed principal components. Pie chart shows how the total variance is split between different task variables. (**d, h**) Dot product between all pairs of the first 15 demixed principal components (upper-right triangle) and correlations between all pairs of the first 10 demixed principal components (bottom-left triangle). Stars denote statistical significance of the two PC axes being significantly non-orthogonal [3] (p<0.001, one-sided paired t-test for $f_1 \cdot f_2 > 3.3\sqrt{N}$, where f_1, f_2 are the respective axes and N the dimension of the the ccRNN cerebral network). Source data are provided as a Source Data file.

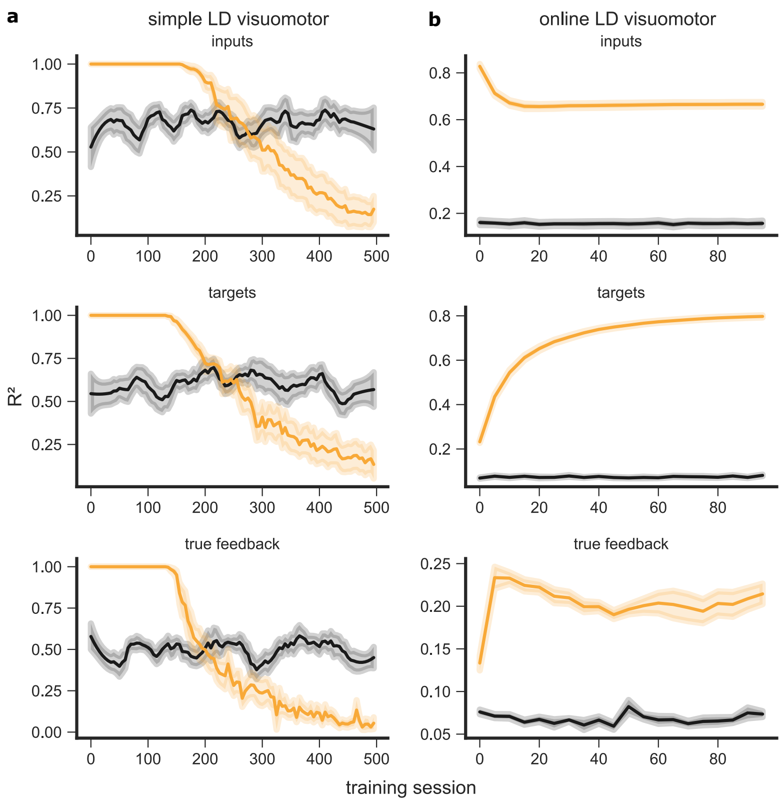

Figure S8. Linear regression over learning determines how much variation of the cerebellar activities are explained by the inputs, targets and true feedback. (**a**) Three linear regression models for input, target and true feedback information during learning of the simple linedrawing visuomotor task (orange) compared to shuffled data (black). (**b**) Same as in (**a**) for the online linedrawing visuomotor task. Error bars represent mean $\pm$ SEM across 10 different initial conditions. Source data are provided as a Source Data file.

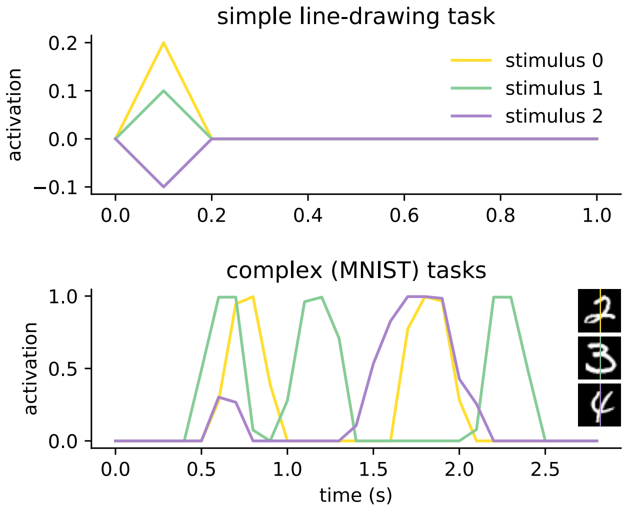

Figure S9. Input activations across time for the simple line drawing (top; cf. Fig. 2) and online MNIST based tasks (bottom; cf. Fig. 3). It is shown for task for 3 example stimuli; for the MNIST based tasks the activation shown is for one example pixel. Stimuli are colour coded as in Figs. 2,3.

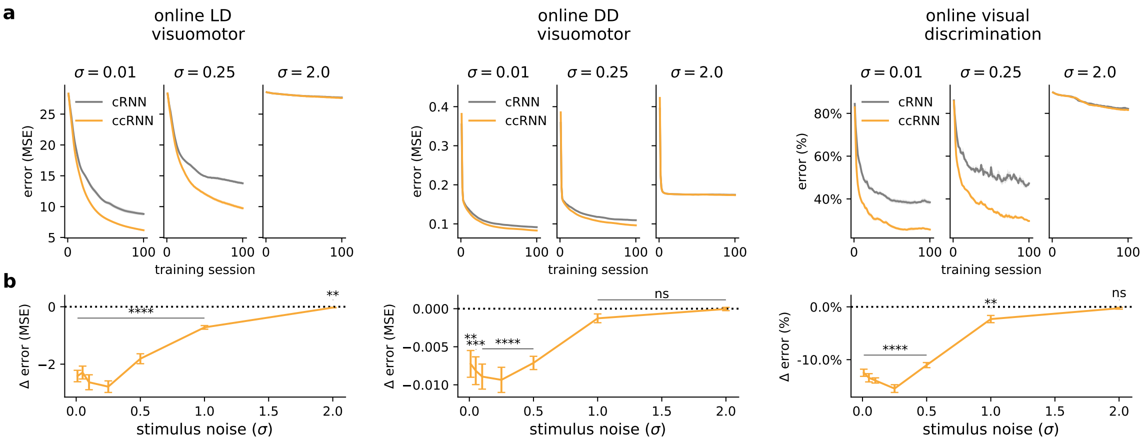

Figure S10. Learning the online LD visuomotor (left), online DD visumotor (middle) and online visual discrimination (right) tasks under varying degrees of input noise ω, where $\omega \sim \mathcal{N}(0, \sigma^2)$. (**a**) Learning curves for low, medium and high levels of noise. (**b**) (Total) training error across different noise levels (ns denotes not significant: online DD visuomotor p=0.052 ($\sigma = 1$), p= 0.865 ($\sigma = 2$); online visual discrimination p=0.153). **: p<0.01, ***: p<0.001, ****: p<0.0001 (two-sided paired t-test between cRNN and ccRNN). Error bars represent mean $\pm$ SEM across 10 different initial conditions. Source data are provided as a Source Data file.

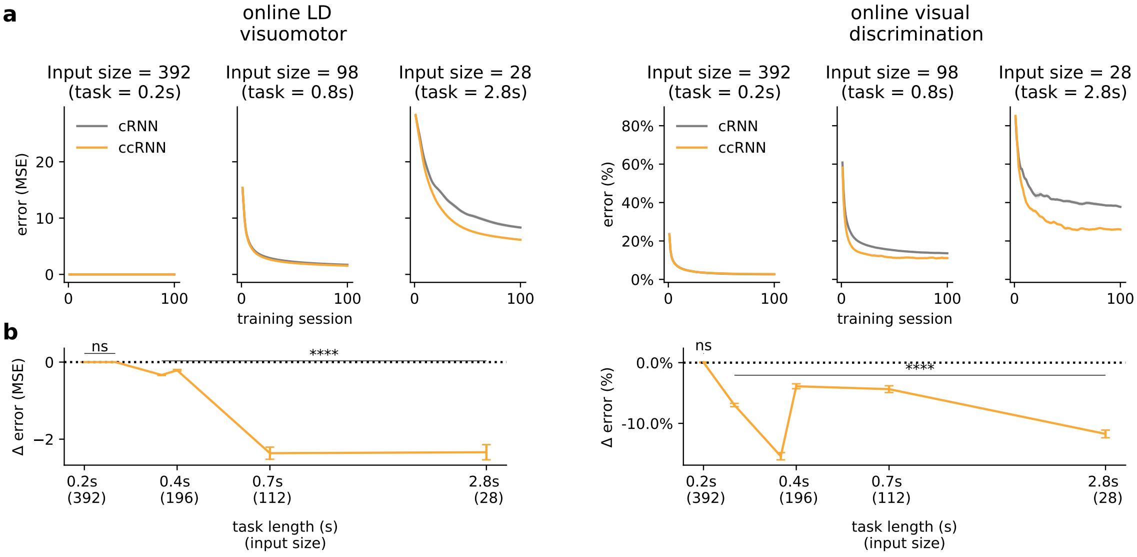

Figure S11. Learning the online LD visuomotor (left) and online visual discrimination (right) tasks for different stimulus (chunks of image) sizes. Since each MNIST image is of total size 784, the total sequence length of the task with input size N can be calculated as $\frac{784}{N} \times \Delta t$ (where $\Delta t = 0.1$s). Note that online DD visuomotor variant is not included here since as the number of timesteps decreases there is not enough time for the model output to construct a digit. (**a**) Learning curves for long (as presented in main text), medium and small task lengths. (**b**) (Total) training error across different input size (and therefore task lengths) values (ns denotes not significant: online LD visuomotor; p=0.093 (0.2s), p= 0.859 (0.4s); online visual discrimination p=0.239). ****: p<0.0001 (two-sided paired t-test between cRNN and ccRNN). Error bars represent mean $\pm$ SEM across 10 different initial conditions. Source data are provided as a Source Data file.

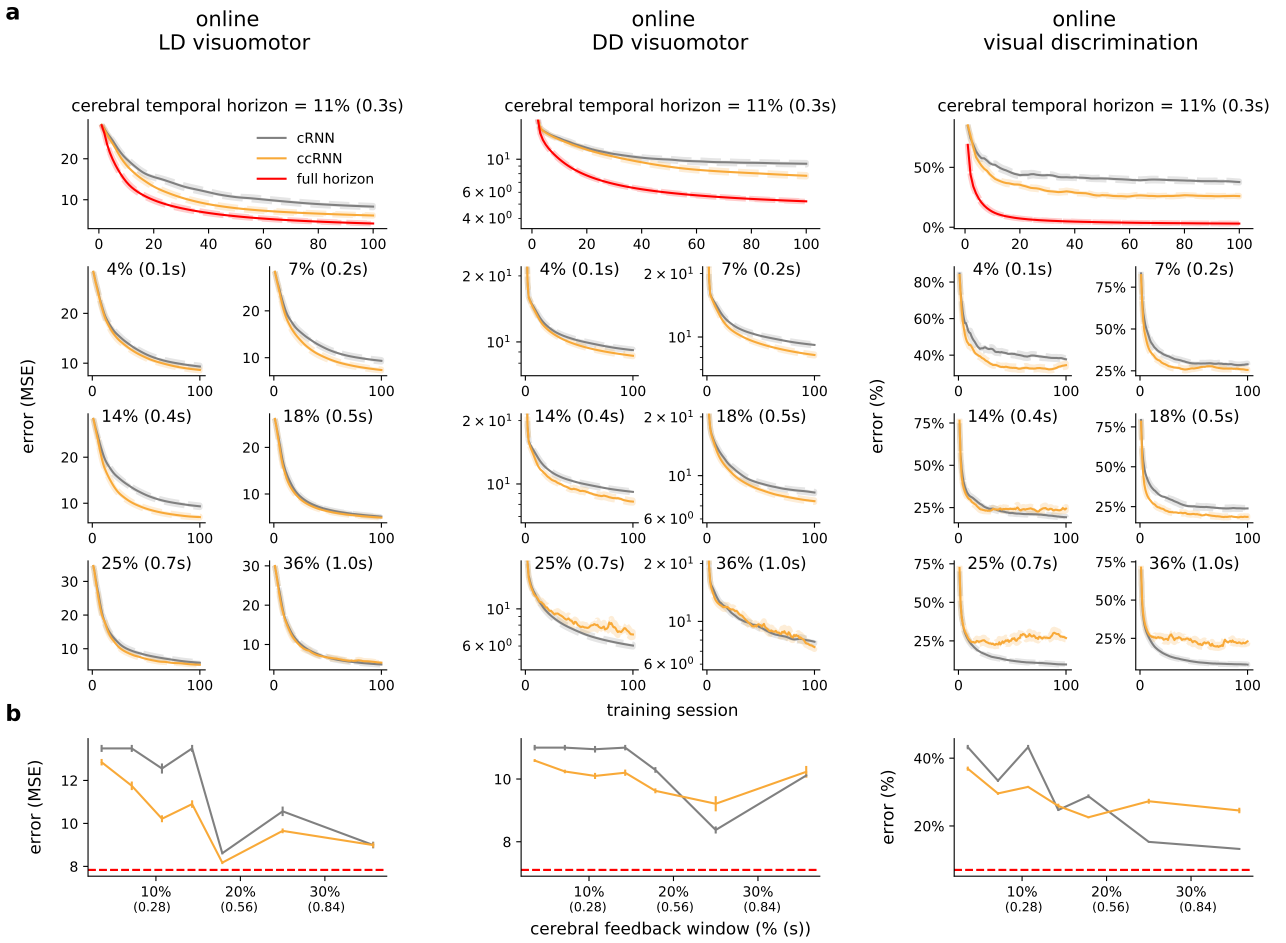

Figure S12. Learning for different cerebral feedback horizons for the online visuomotor and discrimination tasks (cf. Fig. 3d). Feedback horizon is given as percentage of task duration (28 timesteps). (**a**) Learning curves; results presented in main text (Fig. 3b) shown on top row along with RNN trained with full horizon (i.e. cerebral feedback horizon = 100%). (**b**) (total) error during learning across different feedback horizons. Error bars represent mean $\pm$ SEM across 10 different initial conditions. Source data are provided as a Source Data file.

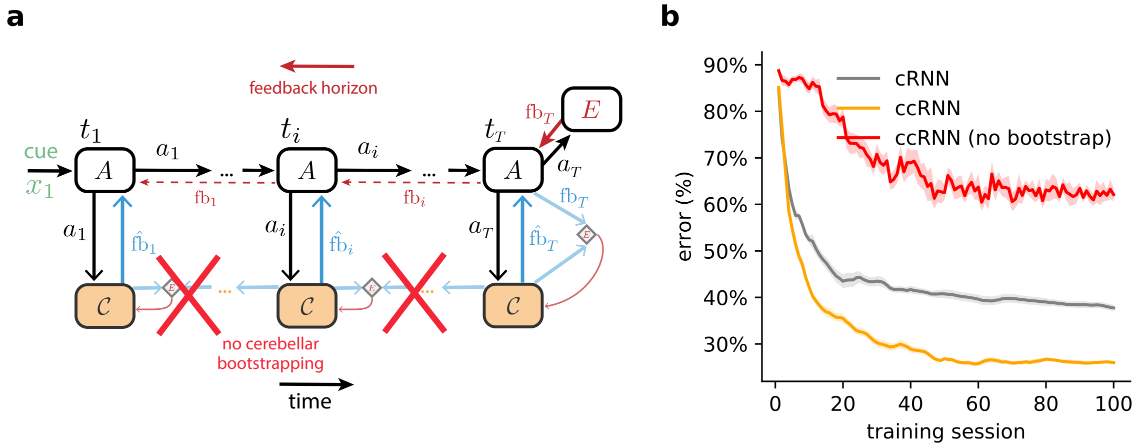

Figure S13. Learning of ccRNN in the online visual discrimination task without bootstrapping. (**a**) By removing the cerebellar bootstrap, the cerebellum no longer uses its own estimates during training (i.e. the right hand side of Eq. 3 is removed). (**b**) Learning curve for the ccRNN with no bootstrapping for the online visual discrimination task. For this task external feedback only comes at the end, so bootstrapping is a critical component for cerebellar learning. Error bars represent mean $\pm$ SEM across 10 different initial conditions. Source data are provided as a Source Data file.

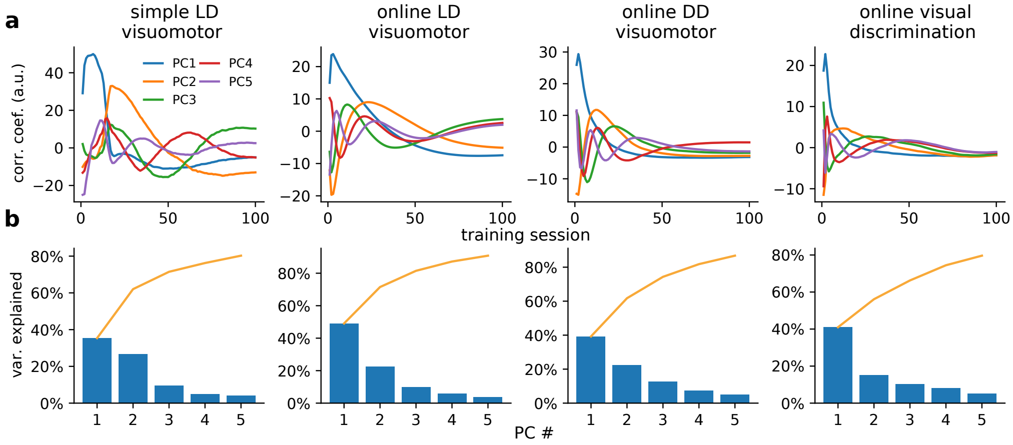

Figure S14. Pair-wise correlations over learning. (**a**) extension of Fig. 6b for top 5 principal components. (**b**) Variance explained by each component (accumulation in orange). Data grouped across 10 different initial conditions, where for each condition we sample 600 active pairs for the simple LD visuomotor task and 1000 active pairs for the online tasks (see Methods). Source data are provided as a Source Data file.

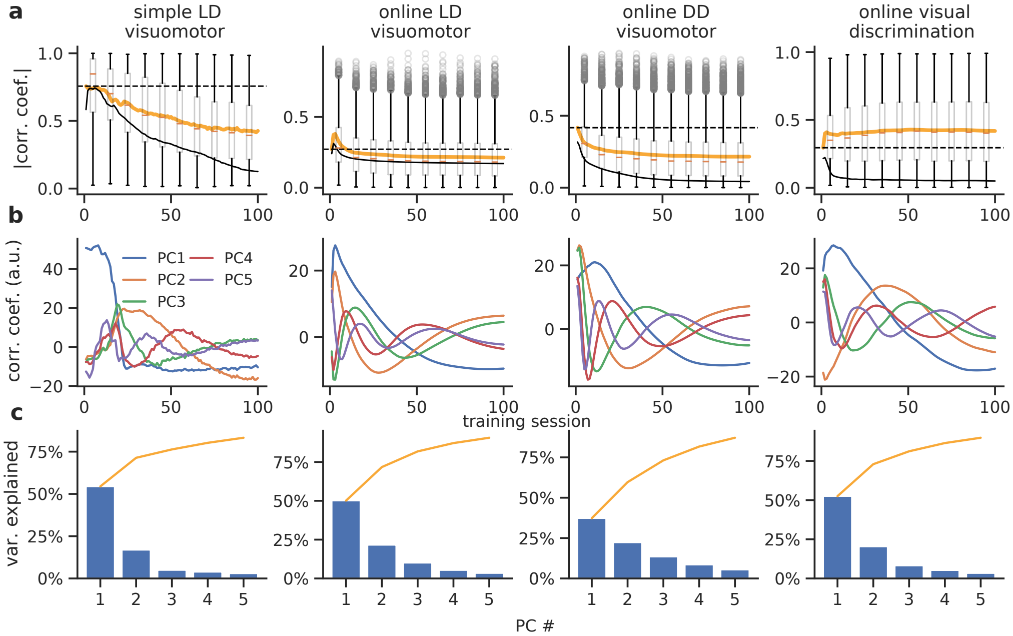

Figure S15. Pair-wise correlations over learning with a *fixed* cerebellar module. (**a**) Box plot showing the mean and distribution of pair-wise cerebro-cerebellar correlations over learning. Mean correlation coefficient for the fully plastic ccRNN model (solid black line) and fully fixed ccRNN (i.e. without any form of plasticity in both cerebral and cerebellar networks; dashed black line) are given for reference. Boxplot shows median (horizontal dark orange line), interquartile range (IQR; box with centre at mean); whiskers show respective quartiles extended by 1.5 $\times$ IQR, where circles denote individual outliers beyond this range. (**b**) Top 5 principal components. (**c**) Variance explained by each component (accumulation in orange). Data grouped across 10 different initial conditions, where for each condition we sample 600 active pairs for the simple LD visuomotor task and 1000 active pairs for the online tasks (see Methods). Source data are provided as a Source Data file.

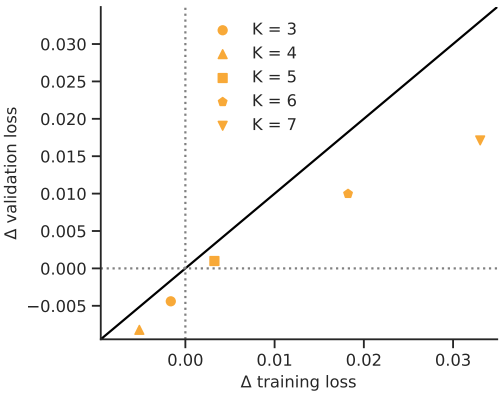

Figure S16. Generalisation of ccRNN (orange scatter) for feedback horizons K from 3 to 7. The change in loss is computed with reference to the cRNN (i.e. ccRNN - cRNN). Training loss is calculated *after* training for a fair comparison with final validation performance. Source data are provided as a Source Data file.

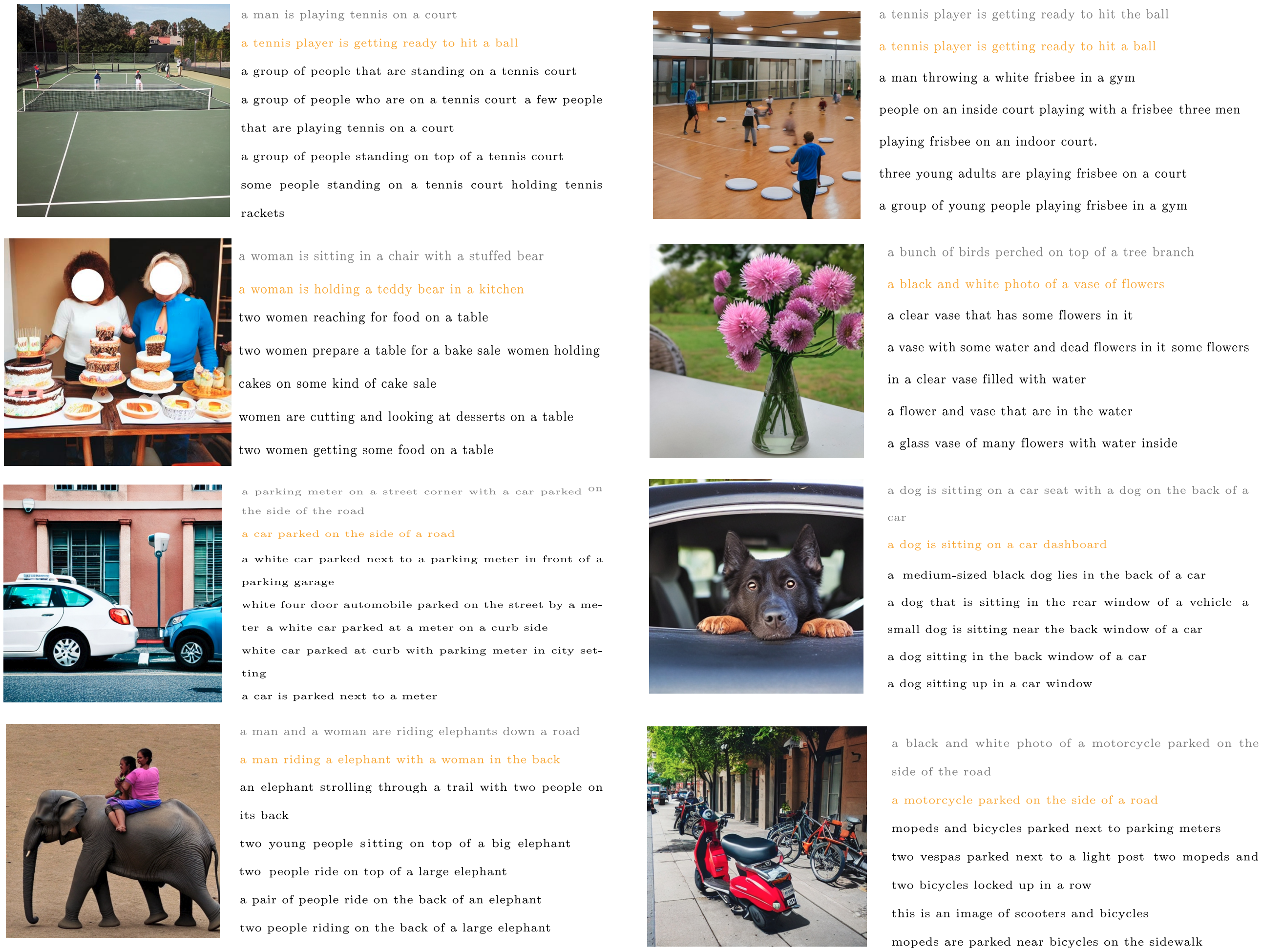

Figure S17. Example images and captions from the validation set with corresponding model captions (cRNN in grey and ccRNN in orange) and gold standard captions (black). Here we show a combination of examples of how the models describe the presented image. In some case all or some models fail to give an accurate description of the image. In other cases all models are able to provide an accurate caption for the image, with each model displaying subtle differences in the generated captions. The images shown here were generated on deepAI.org for illustration purposes only.

References

[1] Max Jaderberg, Wojciech Marian Czarnecki, Simon Osindero, Oriol Vinyals, Alex Graves, David Silver, and Koray Kavukcuoglu. Decoupled neural interfaces using synthetic gradients. In *Proceedings of the 34th International Conference on Machine Learning*, 70, 1627–1635, 2017.

[2] Masao Ito. Control of mental activities by internal models in the cerebellum. *Nature Reviews Neuroscience*, 9, 304–313, 2008.

[3] Dmitry Kobak, Wieland Brendel, Christos Constantinidis, Claudia E Feierstein, Adam Kepecs, Zachary F Mainen, Xue-Lian Qi, Ranulfo Romo, Naoshige Uchida, and Christian K Machens. Demixed principal component analysis of neural population data. *eLife*, 5, 2016.
